# Onset of Microscopic Polyangiitis in Binephrectomied Patient on Chronic Hemodialysis—Case Report

**DOI:** 10.3389/fimmu.2017.00111

**Published:** 2017-02-13

**Authors:** Aleksandar Jankovic, Vesna Maslarevic-Radovic, Petar Djuric, Jelena Tosic-Dragovic, Ana Bulatovic, Nikola Simovic, Milos Mitrovic, Verica Stankovic-Popovic, Vesna Dopudja-Pantic, Snezana Arandjelovic, Nada Dimkovic

**Affiliations:** ^1^Clinical Department for Nephrology with Dialysis, University Medical Center Zvezdara, Belgrade, Serbia; ^2^Clinical Department for Pulmonology, University Medical Center Zvezdara, Belgrade, Serbia; ^3^Institute for Allergology and Immunology, Clinical Center Serbia, Belgrade, Serbia; ^4^Medical Faculty, Belgrade University, Belgrade, Serbia

**Keywords:** microscopic polyangiitis, chronic hemodialysis, binephrectomy, MPO-ANCA, pulmo

## Abstract

**Introduction:**

Microscopic polyangiitis (MPA) is one of the causes of the pulmonary–renal syndrome associated with elevated non-specific markers of inflammation and antineutrophil cytoplasmic autoantibody (ANCA) positivity in 50–75%. *De novo* occurrence of the disease in patients on chronic hemodialysis (HD) has not been described.

**Case presentation:**

We presented patient who developed MPO-ANCA-associated MPA with lung and musculoskeletal involvement after 4 years on regular HD due to bilateral nephrectomy. After excluding the other causes of MPO-ANCA positivity, diagnosis was confirmed even without renal biopsy. Patient received standard immunosuppression therapy and he is still in remission after 27 months.

**Conclusion:**

The onset of immune-mediated disease could be observed even after introduction of renal replacement therapy, which may be a diagnostic problem. Early recognition and traditional immunosuppressive regiment may provide successful outcome.

## Introduction

Microscopic polyangiitis (MPA) is one of the antineutrophil cytoplasmic autoantibody-associated vasculitis (AAV), along with granulomatosis with polyangiitis (GPA; Wegener’s) and eosinophilic granulomatosis with polyangiitis (Churg-Strauss). Antineutrophil cytoplasmic autoantibody (ANCA) may be specific for myeloperoxidase (MPO-ANCA) or for proteinase 3 (PR3-ANCA) (1).

Microscopic polyangiitis is one of the causes of the pulmonary–renal syndrome, besides Goodpasture’s syndrome, systemic lupus erythematosus, and GPA (2). The onset and relapses of systemic diseases in patient with end-stage renal disease are not so common (3). In this case report, we present the patient who developed MPO-ANCA-associated MPA with lung and musculoskeletal involvement after 4 years of regular hemodialysis (HD), and this is, as far as we could found, the first case of MPA onset in this specific population.

## Case Report

A 72-year-old male underwent right nephrectomy in 2006 and 2 years later total bladder cystectomy due to disseminated ureteral transcellular carcinoma. In November 2010, patients’ renal function deteriorated, and after diagnosis of complicated pyelonephritis, he underwent left-side nephrectomy. Since then he was on chronic HD. He also has a history of arterial hypertension and smoking.

In June 2014, he was hospitalized due to swollen and painful wrists, proximal interphalangeal and metacarpophalangeal joints, and subfebrile temperature. Physical examination revealed lowered right basilar respiratory ton and systolic precordial murmur. Laboratory findings at admission revealed high CRP level and low hemoglobin level, despite erythropoietin therapy and iron supplementation (Table 1). Arterial blood gases pointed out acute partial respiratory insufficiency (pO_2_ 7.3 kPa, pCO_2_ 3.4 kPa, O_2_ saturation 90%). Chest radiography and computed tomography (CT) have shown bilateral pleural effusion (more pronounced in the right side) with subsequent compressive atelectasis and possible consolidation in lower right lung. Diagnostic pleural puncture confirmed exudation (protein level 33 g/L). *Klebsiella*–*Enterobacter* was isolated from the sputum culture, and patient is understood to have respiratory infection. Echocardiography revealed mild aortic stenosis and pericardial effusion (10 mm), without endocardial vegetations. Abdominal ultrasound was insignificant, apart form the missing kidneys. Other tests were performed in order to explain clinical status and high CRP level including bacteriology (hemocultures, coprocultures, nasal and pharyngeal swabs), tumor markers (prostate-specific antigen, carcinoembryonic antigen, CA 19-9, AFP), and markers of viral hepatitis (hepatitis B surface antigen, anti-HCV, anti-HIV), and they all were negative. Esophagogastroduodenoscopy and total colonoscopy were done, and results indicated mild erosive gastritis and diverticulosis sigmae.

**Table 1:** **Biochemical and blood count analysis at hospital admission**.

Analysis	Value	Analysis	Value
Glucose	5.1 mmol/L	Uric acid	359 μmol/L
Cholesterol	3.79 mmol/L	Urea	23.3 mmol/L
HDL cholesterol	0.64 mmol/L	Creatinine	735 μmol/L
LDL cholesterol	1.82 mmol/L	CK	11 U/L
Triglycerides	2.93 mmol/L	Fibrinogen	**6**.**9 g/L**
Albumin	31 g/L	Fe	2.1 μmol/L
protein	66 g/L	UIBC	20.8 μmol/L
Total bilirubin	6.8 μmol/L	TIBC	22.9 μmol/L
Direct bilirubin	1.0 μmol/L	Transferrin saturation	0.09
AST	12 U/L	Potassium	5.3 mmol/L
ALT	11 U/L	Sodium	137 mmol/L
ALP	31 U/L	Phosphorus	1.38 mmol/L
GGT	16 U/L	Bicarbonates	21 mmol/L
LDH	393 U/L	CRP	**355**.**6 mg/L**

**Complete blood count**	**Value**	**Leukocytic formula**	**%**

Leukocytes	9.95 × 10^9^	Neutrophils	78.5
Erythrocytes	3.02 × 10^12^	Lymphocytes	8.0
Hemoglobin	8.8 g/dL	Monocytes	2.3
Hematocrit	0.29	Eosinophils	11.0
MCV	94.7 fL	Basophils	0.2
Platelets	284 × 10^9^		

*Bold font indicates the most important findings in this patients’ blood analysis*.

**Figure 1:**
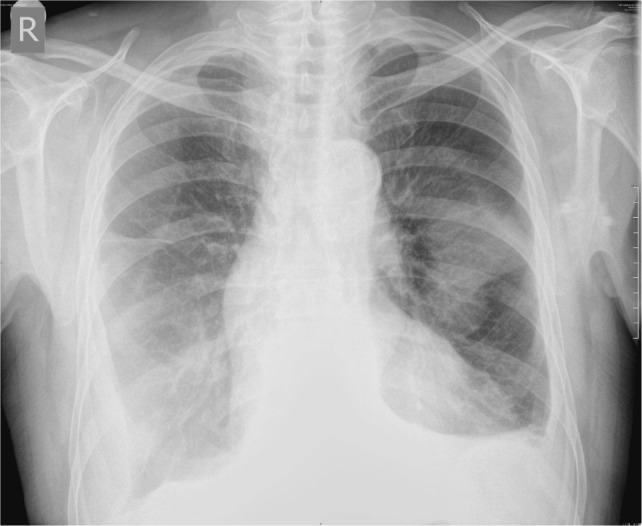
**Initial chest radiography**.

Antibiotic therapy according to sputum antibiogram (Meropenem) was started together with non-steroid and anti-inflammatory drug. After 14 days of antibiotic therapy, joints were still swollen and painful, CRP level was still high (139.3 mg/L), and chest radiography revealed infiltrations in middle and upper zones of the right lung and middle zone of the left lung (Figure 1). We performed respiratory function assessment which pointed out restrictive pattern of respiratory disorder [spirometry: forced expiratory volume in 1 s (FEV1) 55%, FVC 56%, FEV1/FVC 101%], and lung diffusion capacity (DLCO) was 59% of anticipated and pulmonary CT angiography ruled out pulmonary thromboembolism. Results of serology analysis confirmed high levels of anti-MPO antibodies and perinuclear antineutrophil cytoplasmic antibody (p-ANCA) positivity (Table 2).

**Table 2:** **Serology analysis**.

Analysis		Value
Serology		
	ANA (IIF)	1:40
	p-Antineutrophil cytoplasmic autoantibody (ANCA) (IIF)	**1:640**
	Anti-MPO at (ELISA)	**170.7 RU/ml (normal: <20 RU/mL)**

*Bold font indicates the most important findings in this patients’ blood analysis*.

**Figure 2:**
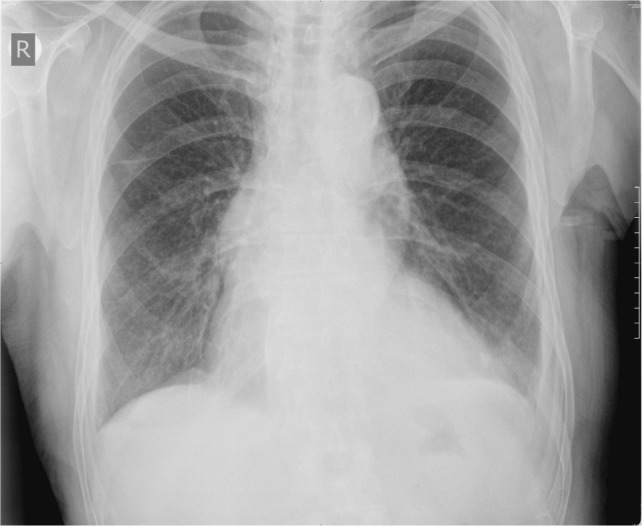
**Chest radiography after 3 months of treatment**.

Since we could not perform kidney biopsy but have strongly positive MPO-ANCA, musculoskeletal and lung symptoms, as well as ruling out other reasons for ANCA positivity, diagnosis of MPA was established (Table 3). Specific induction therapy with intravenous cyclophosphamide (six monthly doses of 800 mg) and intravenous methylprednisolone (six monthly pulses with 3 days of 1 g) was prescribed, followed by oral prednisone (0.5 mg/kg). After 1 month, there were no joint symptoms, CRP level was almost normal (9.8 mg/L), and arterial blood gases were recovered, and after 3 months, chest radiography was normal (Figure 2). After 6 months, control MPO-ANCA was 0.62 RU/mL and p-ANCA 1:80.

**Table 3:** **Clinical manifestations of microscopic polyangiitis, overall prevalence, and findings in our patient [adapted according to Ref. (2, 4, 5)]**.

	Our patient
**Constitutional manifestations**	
Fever (55%)	X
Malaise, fatigue, flu-like syndrome	X
Weight loss (72%)	X
**Other manifestations of microscopic polyangiitis**	
Rapidly progressive glomerulonephritis (80–100%)	
Pulmonary involvement (alveolar hemorrhage, infiltrates, pleural effusion, pleuritis, interstitial fibrosis) (25–55%)	X
Cardiovascular (chest pain, symptoms of heart failure, pericarditis)	
Gastrointestinal manifestations (abdominal pain, gastrointestinal bleeding, colonic ulcerations) (21–58%)	
Neurologic manifestation	
Peripheral nervous system: mononeuritis multiplex, distal symmetrical polyneuropathy (37–72%)	
Central nervous system: cerebral hemorrhage, pachymeningitis, non-hemorrhagic cerebral infarctions (17–30%)	
Musculoskeletal involvement (arthritis, arthralgia, myalgia)	X
Skin manifestations (palpable purpura, livedo reticularis, nodules, urticaria, and skin ulcers with necrosis) (30–60%)	

Patient is still in stable remission after 27 months of induction therapy followed by maintenance therapy (oral azathioprine 100 mg/day).

## Discussion

This case report presents the patient who developed MPO-ANCA-associated MPA with lung and musculoskeletal involvement after 4 years on regular HD.

Incident MPA patients are predominantly males (1.8:1.0) with an average age of onset between 50 and 60 years and with predominant increase of non-specific markers of inflammation (erythrocyte sedimentation rate and C reactive protein) and normochromic, normocytic anemia (4). Our patient was older, but he had characteristic laboratory findings. There is no test that has high diagnostic specificity for MPA, since ANCA is detected in 50–75% of patients. Among them, 90–95% have perinuclear staining pattern (p-ANCA) caused by antibodies against myeloperoxidase (MPO-ANCA) (2, 4). Therefore, renal biopsy is of a great diagnostic value.

In this patient, there were several diagnostic obstacles. Kidneys, as the most frequently involved organs, were missing so biopsy was not feasible. Also, it is unusual to expect the onset of systemic disease in immunocompromised uremic patients who also have very rare relapses of previously diagnosed immune-mediated disease (3). In addition, we had to exclude the other reasons of MPO-ANCA positivity: inflammatory bowel diseases, rheumatoid arthritis, infections (endocarditis, tuberculosis, and amebiasis), drug-induced vasculitis (hydralazine, propylthiouracil), and malignancy (6–13). Also, patients on HD may have elevated p-ANCA or c-ANCA if hemodiafiltration is used, but our patient was on standard bicarbonate dialysis (14). We also had clinical signs which correlated with these serological findings and finally, after 6 months of immunosuppression therapy, p-ANCA level was almost normal.

After the diagnosis of MPA was set, in agreement with pulmonologist and immunologist, traditional and most frequently used treatment with monthly intravenous cyclophosphamide and methylprednisolone followed by oral prednisone was introduced. This regimen is known to lower mortality in AAV (2, 15). Good response to therapy justifies our diagnosis and patient is in stable remission after 27 months.

## Concluding Remarks

In conclusion, the onset of immune-mediated disease could be observed even after introduction of renal replacement therapy, which may be a diagnostic problem. Early recognition and traditional immunosuppressive regiment may provide successful outcome.

The patient has given his written, informed consent to publish the information appearing in the article.

## Author Contributions

AJ and ND have designed the paper; VM-R, PD, JT-D, AB, NS, MM, and VS-P have been part of every step in this patients’ very hard and complicated diagnostic and therapeutic course and give valuable interpretation of data. All the coauthors revised paper critically and gave final approval of this version for publishing. They have ensured that all aspects of the work are accurate and have been appropriately investigated and resolved. VD-P and SA were our consultants (pulmonolgist and immunologist).

## Conflict of Interest Statement

The authors declare that the research was conducted in the absence of any commercial or financial relationships that could be construed as a potential conflict of interest.

## Abbreviations

MPA, microscopic polyangiitis; ANCA, antineutrophil cytoplasmic autoantibody; HD, hemodialysis; RRT, renal replacement therapy; MPO, antineutrophil cytoplasmic autoantibody specific for myeloperoxidase; PR3, antineutrophil cytoplasmic autoantibody specific for proteinase 3; AAV, antineutrophil cytoplasmic autoantibody-associated vasculitis; GPA, granulomatosis with polyangiitis; EGPA, eosinophilic granulomatosis with polyangiitis; SLE, systemic lupus erythematosus; ESRD, end-stage renal disease; TCC, transcellular carcinoma; PIP, proximal interphalangeal; MCP, metacarpophalangeal; CRP, C reactive protein; pO_2_, partial pressure of oxygen in arterial blood; pCO_2_, partial pressure of carbon dioxide in arterial blood; O_2_, oxygen; CT, computed tomography; PSA, prostate-specific antigen; CEA, carcinoembryonic antigen; CA 19-9, cancer antigen 19-9; AFP, alpha-fetoprotein; HBsAg, hepatitis B surface antigen; anti-HCV, anti-hepatitis C antibody; anti-HIV, anti-human immunodeficiency virus antibody; NSAID, non-steroid and anti-inflammatory drug; FEV1, forced expiratory volume in 1 s; FVC, forced volume vital capacity; FEV1/FVC, FEV1/FVC ratio; DLCO, diffusing capacity for carbon monoxide; p-ANCA, perinuclear antineutrophil cytoplasmic antibody; c-ANCA, cytoplasmic antineutrophil cytoplasmic antibody.
